# Radiative Transfer Modeling of Phytoplankton Fluorescence Quenching Processes

**DOI:** 10.3390/rs10081309

**Published:** 2018-08-20

**Authors:** Peng-Wang Zhai, Emmanuel Boss, Bryan Franz, P. Jeremy Werdell, Yongxiang Hu

**Affiliations:** 1 Department of Physics, University of Maryland Baltimore County, Baltimore, MD 21250, USA; 2 School of Marine Sciences, University of Maine, Orono, ME 04401, USA; emmanuel.boss@maine.edu; 3 NASA Goddard Space Flight Center, Code 616, Greenbelt, MD 20771, USA; bryan.a.franz@nasa.gov (B.F.); jeremy.werdell@nasa.gov (J.W.); 4 MS 475 NASA Langley Research Center, Hampton, VA 23681-2199, USA; yongxiang.hu-1@nasa.gov

**Keywords:** radiative transfer, ocean optics, inelastic scattering, fluorescence

## Abstract

We report the first radiative transfer model that is able to simulate phytoplankton fluorescence with both photochemical and non-photochemical quenching included. The fluorescence source term in the inelastic radiative transfer equation is proportional to both the quantum yield and scalar irradiance at excitation wavelengths. The photochemical and nonphotochemical quenching processes change the quantum yield based on the photosynthetic active radiation. A sensitivity study was performed to demonstrate the dependence of the fluorescence signal on chlorophyll a concentration, aerosol optical depths and solar zenith angles. This work enables us to better model the phytoplankton fluorescence, which can be used in the design of new space-based sensors that can provide sufficient sensitivity to detect the phytoplankton fluorescence signal. It could also lead to more accurate remote sensing algorithms for the study of phytoplankton physiology.

## 1. Introduction

Solar induced fluorescence of chlorophyll a in phytoplankton is an important source of information on phytoplankton biomass variation [1,2]. Chlorophyll a concentration is a primary factor that influences the fluorescence signal [3], however, the signal is also strongly impacted by phytoplankton health (or physiology) as well as environmental and physical factors such as species composition, pigment packaging, nonphotochemical quenching (NPQ), and the availability of light and nutrients (notably biologically available iron) in the ocean surface layer [3–5]. A variety of algorithms were developed to study phytoplankton physiological state from satellite observations of chlorophyll a fluorescence [3,5–9].

To understand the fluorescence signal emanating from the ocean, it is important to have a rigorous radiative transfer model that is capable of simulating fluorescence strength in response to different physiological and environmental factors. In recent years, several approximate analytical models [10,11] and rigorous radiative transfer models [12–19] were developed to predict inelastic scattering processes in ocean waters, which included the methods of Monte Carlo [12,13], invariant embedding [14], matrix operator [15], successive order of scattering [16,17], and discrete ordinates [18,19]. The main inelastic scattering mechanisms considered in these works were Raman scattering, fluorescence by dissolved organic matter (FDOM), and fluorescence by chlorophyll a phytoplankton [14]. There remains, however, a lack of radiative transfer models that can account for the photo-compensation mechanisms [8]. This paper reports the first radiative transfer model that can simulate the impacts of both photochemical and nonphotochemical quenching effects on the phytoplankton fluorescence signal within the ocean and that is observed with space-based sensors. The primary inputs of the model are the inherent optical properties of the atmospheric and oceanic components and the physiological parameters which fully describe the fluorescence quantum yield as a function of spectral scalar irradiance in ocean waters.

Our model can be used to perform systematic studies of fluorescence in response to environmental factors and ocean water inherent optical properties, which in turn leads to better and more accurate remote sensing algorithms. The paper is organized in the following way. Section 2 outlines the theoretical implementation of photo-compensation in a radiative transfer model; Section 3 presents representative simulations on fluorescence in response to different atmosphere and oceanic conditions; Section 4 discusses a few limitations and some potential applications of our model; Section 5 summarizes the main conclusion.

## 2. Methods

Our radiative transfer model is based on the successive order of scattering (SOS) method for coupled atmosphere and ocean systems [20,21]. Recently inelastic scattering in ocean waters was integrated in the SOS code [16,17,22]. This paper uses the theoretical formulation in [17] for the implementation of inelastic scattering in radiative transfer. A key parameter which determines the fluorescence strength is the quantum yield Φ_*C*_, which typically ranges between 0.01 and 0.05, and depends on phytoplankton species, physiological state, and the inherent optical properties of the water column [1,3,8,14]. We used a constant number for the quantum yield in Zhai et al. [17] to demonstrate the sensitivity of fluorescence on environmental parameters, which is applicable for a wide range of waters [6].

To account for the fluorescence quenching processes in the radiative transfer, we adopted here the model for quantum yield developed by Morrison et al. [1,8]:

(1) $$\Phi_{C}=q_{I}e^{-\text{IPAR}/E_{T}}\left(\Phi_{Cmin}e^{-\text{IPAR}/E_{k}}+\Phi_{Cmax}\left(1-e^{-\text{IPAR}/E_{k}}\right)\right),$$

where the parts in and outside of the parentheses represent photochemical and nonphotochemical quenching, respectively; *q*_*I*_ is a parameter between 0 and 1 which stands for thermal dissipation of excess excitation energy through reaction center quenching. Alternatively, *q*_*I*_ can be viewed as the relative probability of a photon absorbed by pigment molecules being delivered to a photosystem II reaction center [1,8]. IPAR is the instantaneous photosynthetically available radiation, which was obtained from the scalar irradiance *E*_*o*_ via [23]:

(2) $$\text{IPAR}=\int_{\lambda_{1}}^{\lambda_{2}}E_{o}(\lambda )\frac{\lambda }{hc}d\lambda ,$$

(3) $$E_{o}(\lambda )=\int_{4\pi }I(\Omega ,\lambda )d\Omega ,$$

where the symbol Ω represents the solid angle; and I is the radiance. In the IPAR definition, *λ*_1_ = 400 nm and *λ*_2_ = 700 nm are normally used for the lower and higher wavelength limits. In this work, we adopted *λ*_1_ = 370 nm and *λ*_2_ = 690 nm to be consistent with the inelastic fluorescence source term limits (see Equation (4) below) [14,17]. The different limits are not expected to have significant impacts to our analysis and conclusions in this paper.

*E*_*k*_ and *E*_*T*_ are the light saturation parameters for photochemical and energy-dependent quenching, respectively; Φ_*Cmin*_ and Φ_*Cmax*_ are the minimum and maximum quantum yields, respectively. In our radiative transfer model, *q*_*I*_, *E*_*T*_, *E*_*k*_, Φ_*Cmin*_, and Φ_*Cmax*_ are all input parameters, which may vary considerably depending on phytoplankton species and physiological states. *E*_*o*_ and IPAR are calculated by the radiative transfer solver, given the solar spectral irradiance and inherent optical properties of the atmosphere and oceanic constituents.

First we run the radiative transfer solver through the whole range of excitation wavelengths to determine the scalar irradiance *E*_*o*_ at different depths. IPAR is obtained through Equation (2). Then the quantum yield is calculated from Equation (1). The fluorescence scattering coefficient *b*_*C*_ is determined by [14,17]:

(4) $$b_{C}\left(z,\lambda ,\lambda_{e}\right)=a_{p}\Phi_{C}\frac{1}{\sqrt{2\pi }\sigma_{C}}\text{exp}\left[-\frac{{\left(\lambda -\lambda_{C,0}\right)}^{2}}{2\sigma_{C}^{2}}\right]\frac{\lambda_{e}}{\lambda },\;370\text{nm}<\lambda_{e}<690\text{nm}$$

where *z* denotes the vertical position; *λ* and *λ*_*e*_ are the emission and excitation wavelengths, respectively; *a*_*p*_ is the phytoplankton absorption coefficient; *λ*_*C*,0_ = 685 nm is the wavelength of maximum emission; and *σ*_*C*_ = 10.6 nm is the Gaussian standard deviation [14]. The source function *S*_*C*_ at emission wavelength for fluorescence is [14,17]:

(5) $$S_{C}(z,\lambda )=\frac{1}{4\pi }\int_{0}^{\infty }b_{C}\left(z,\lambda ,\lambda_{e}\right)\cdot E_{o}\left(z,\lambda_{e}\right)d\lambda_{e},$$

which assumes that the fluorescence emission is isotropic [24].

The plane-parallel inelastic vector radiative transfer equation without thermal emission is:

(6) $$\mu \frac{dL(z,\mu ,\phi ,\lambda )}{dz}=-c(z,\lambda )L(z,\mu ,\phi ,\lambda )+S\left(z,\mu_{0},\mu ,\phi ,\lambda \right)+S_{i}\left(z,\mu_{0},\mu ,\phi ,\lambda \right),$$

where *μ*_0_ = cos *θ*_0_; *μ* = cos *θ*; *θ*_0_ and *θ* are the solar and viewing zenith angles, respectively; *ϕ* is the viewing azimuth; **L** = [I, Q, U, V]^T^; I, Q, U, V are the Stokes parameters; and superscript T stands for transpose; **S** and **S**_*i*_ are the elastic and inelastic source terms, respectively; *c* is the extinction coefficient, also called beam attenuation coefficient in ocean optics The inelastic source term is the summation of three mechanisms:

(7) $$S_{i}=S_{R}+S_{Y}+S_{C},$$

where the subscripts *R*, *Y*, and *C* stand for Raman scattering, FDOM, and chlorophyll fluorescense, respectively. The fluorescence source matrix **S**_*C*_ is assumed to be independent of polarization so that **S**_*C*_ = [*S*_*C*_, 0, 0, 0]^T^, where *S*_*C*_ is given by Equation (5). The source function **S**_*R*_ and **S**_*Y*_ are the same as in [17]. With both elastic and inelastic source terms are known, the radiative transfer solver is used at emission wavelengths to find the total radiation field.

## 3. Results

We used the model to simulate the fluorescence signals for sensors located at both the top of the atmosphere (TOA) and the top of the ocean (TOO). The simulation was for a hypothetical hyperspectral sensor similar to the ocean color instrument onboard NASA’s next generation satellite: the Plankton, Aerosol, Cloud and ocean Ecosystem mission (PACE) [25]. The light field at the emission wavelength depends on the inherent optical properties at both the emission and excitation spectral ranges. Therefore the inherent optical properties of aerosols and hydrosols used in the simulation need to be spectrally consistent and realistic.

It was assumed that the inherent optical properties of the ocean are described using three components: pure sea water, colored dissolved organic matter (CDOM), and particles with properties covariant with the pigment chlorophyll a concentration [Chla], which are referred as the [Chla] covariant particles hereafter. Both pure sea water and the [Chla] covariant particles scatter and absorb light, while CDOM is assumed to absorb only. The absorption of pure sea water in Zhai et al. [17] was based on Pope and Fry [26]. In this work, we updated the absorption coefficient of pure sea water using a new dataset by Lee et al. [27]. The scattering coefficient of pure sea water is from [14,28]. The absorption coefficient of CDOM decays exponentially as wavelength increases with a decay constant of 0.018 nm^−1^ [17,29,30]. The absorption coefficient for the [Chla] covariant particles follows the parameterization in Bricaud et al. [31], which represents an average behavior of phytoplankton absorption with pigment packaging included. The extinction coefficient of the [Chla] covariant particles is the same as in Zhai et al. [17] (see Equation (16)), which is based on Voss et al. [32] and the International Ocean Color Coordinating Group (IOCCG) report [30]. The scattering coefficient is modeled as the difference between the extinction and absorption coefficients. The phase function of the ocean water is a weighted sum of the pure ocean water[14] and the [Chla] covariant particles, which is determined by the backscattering ratio (see Equations (18) and (19) in Zhai et al. [17]). The ocean water scattering Mueller matrix is the average ocean water measurement by Voss and Fry [33].

The atmosphere is a mixture of aerosols and molecules with molecular number density determined by the 1976 US standard atmosphere [34]. The depolarization of 0.0284 is used to in the calculation of the Rayleigh scattering matrix [35]. The aerosol scattering matrix follows the maritime aerosol with a humidity of 80% [36], and the aerosol vertical distribution is the average height distribution in [37]. The aerosol and Rayleigh scattering matrix are internally mixed by using their scattering coefficients as weighting factors [38]. Gas absorption has been incorporated, which includes ozone, oxygen, water vapor, nitrogen dioxide, methane, and carbon dioxide. First, the atmospheric radiative transfer simulator (ARTS) [39] and the HITRAN2012 database [40] are used to build a hyperspectral lookup tables for gas absorption coefficients of water vapor, oxygen, carbon dioxide, and methane. Absorptions by ozone and nitrogen dioxide are included separately based on the measurements in [41] and [42]. respectively. A hypothetical Gaussian instrument line shape (ILS) function is assumed with full width at half maximum of 5 nm. Within each spectral interval of 5 nm, the radiance is assumed to be an exponential function of the gas absorption optical depth so that we can solve a few wavelengths to fit the hyper-spectral variation due to gas absorption, which is then convolved with the ILS to simulate the response of a satellite sensor. This philosophy is similar to the double–k method [43]. The solar irradiance spectra from [44] is used as incident source of the radiative transfer system.

To simulate the quenching processes, the parameters in Equation (1) need to be assigned. We used *q*_*I*_ = 0.35, Φ_*Cmin*_ = 0.03, Φ_*Cmax*_ = 0.09, *E*_*k*_ = 55 μmol quanta m^−2^s^−1^, *E*_*T*_ = 634 μmol quanta m^−2^s^−1^. These values are consistent with the findings from [8], though we recognized they are variable for different phytoplankton populations. In addition to the chlorophyll fluorescence, the Raman scattering and FDOM were also included in the simulation using the same scheme as [17]. This includes chlorophyll a concentration [Chla] = 0, 0.03, 0.1, 1, 10 mg/m^3^; aerosol optical depth at 550 nm *τ*_*a*_ = 0, 0.1, 0.2, 0.5; and solar zenith angle *θ*_*s*_ = 0, 30, 60, 78°. It is more convenient to study the radiance field in terms of reflectance, which is defined as:

(8) $$\rho (z,\theta ,\phi )=\pi \cdot \frac{I(z,\theta ,\phi )}{E_{d}(z)}$$

where *I* is the radiance and *E*_*d*_ is the downwelling irradiance.

**Figure 1.**
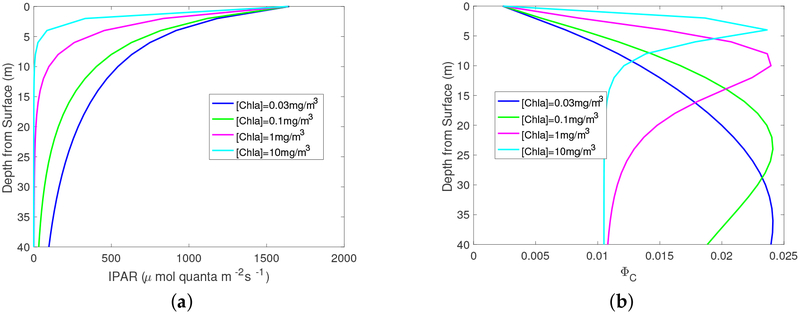
(a) IPAR and (b) quantum yield as a function of depth for a set of [Chla]. The aerosol optical depth at 550 nm is 0.1 and the solar zenith angle is 30°.

Equation (1) builds a connection between the quantum yield and IPAR. It is informative to show how IPAR changes with depths in water and how the quantum yield responds to this variation. Figure 1 shows IPAR and the quantum yield as a function of depth measured from the water surface. For all [Chla] values, IPAR is approximately 1500 μmol quanta m^−2^s^−1^ at the surface, as IPAR is dominated by the atmospheric condition, which is kept the same in these simulation cases. As the depth increases, IPAR decreases faster for larger [Chla] values. As a consequence, the quantum yield starts with the same value at the surface, and increases to a maximum at different levels, and decreases again after that, for different [Chla] values. The vertical location of the peak quantum yield values is deeper for lower [Chla] values. The fluorescence signal at the ocean surface and TOA is essentially a vertical integration of the quantum yield, scalar irradiance, and particle absorption coefficients as shown in Equations (4)–(6). A higher location of the quantum yield peak indicates a stronger fluorescence signal, even though the minimum and maximum values of quantum yield are generally the same as suggested by Figure 1b.

**Figure 2.**
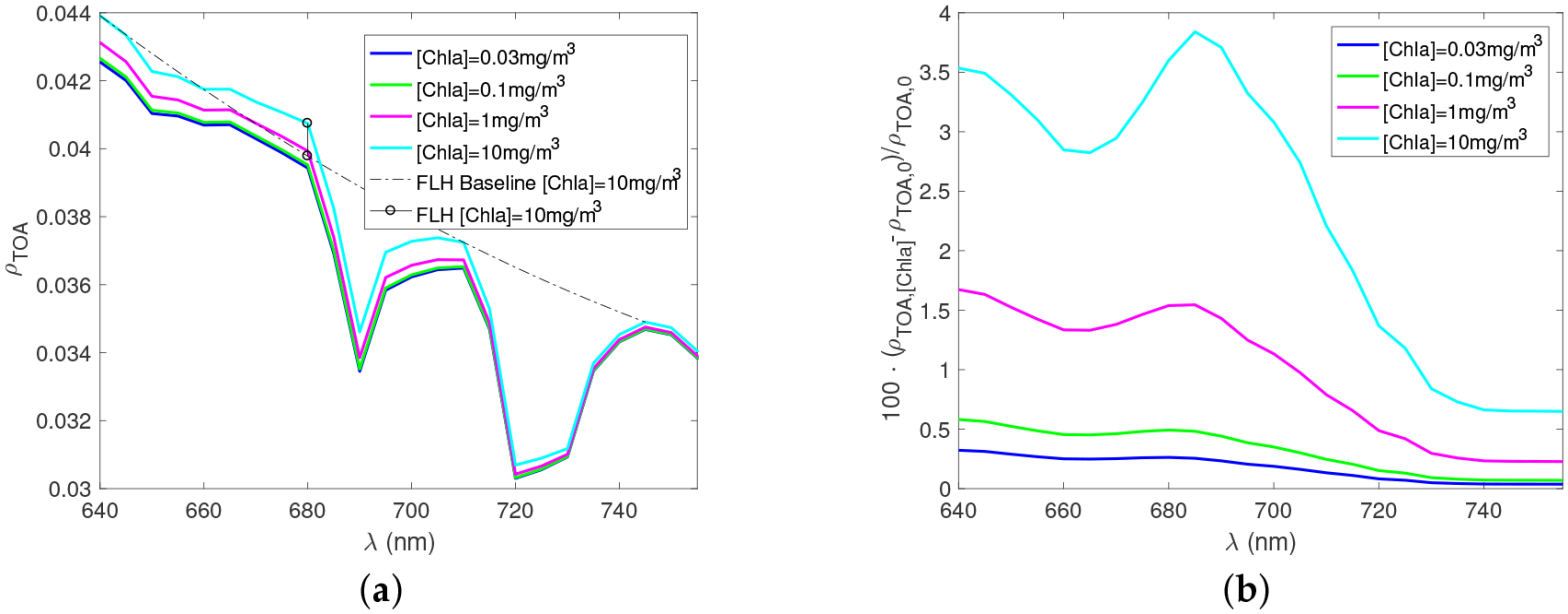
(**a**) The nadir viewing reflectance at TOA as a function of wavelengths for the cases shown in Figure 1. The dash line shows the baseline determined by measurements at 640 nm, 710 nm, and 745 nm for [Chla] = 10 mg/m^3^. The solid line with open circle symbols shows the magnitude of fluorescence line height relative to the baseline for [Chla] = 10 mg/m^3^. (**b**) The percentage ratio of fluorescence signals in the total reflectance observed at TOA.

Figure 2a shows the TOA reflectance *ρ*_TOA_ viewing at nadir (*θ* = 0° and *ϕ* = 0°) as a function of wavelength from 640 to 750 nm. Gas absorption features due to oxygen B band centered around 690 nm and water vapor centered around 650 nm and 720 nm are clearly seen. The fluorescence signal centered at 685 nm increases as chlorophyll a concentration increases. Figure 2b shows the relative change of reflectances *ρ*_TOA,[Chla]_ with respect to *ρ*_TOA,0_, where the subscript 0 stands for [Chla] = 0 mg/m^3^. The peak value of relative change varies between 0.2% to 3.8%, which can be detected by ocean color sensors. The requirements for advanced ocean radiometers specify a signal to noise ratio of 1000 in the visible [45], which could theoretically detect signal variations at a level of 0.1%.

The fluorescence line height (FLH) algorithm developed for the NASA Moderate Resolution Imaging Specroradiometer (MODIS) calculates the baseline signals at 678 nm from measurements at 667 nm and 748 nm [46,47]. The algorithm was a compromise of available bands that sometimes lead to negative FLH [46]. In future satellite missions that measure hyperspectral radiances, fluorescence line height algorithms can be designed using optimal combination of wavelengths. In Figure 2a, we used the TOA reflectance at 640 nm, 710 nm, and 745 nm to obtain a second order polynomial fitting of the baseline signal level without fluorescence for the [Chla] = 10 mg/m^3^ case. The gas absorptions are minimal (column transmittance >97%) at these wavelengths. The peak of the fluorescence are near 680 nm because the signal at 685 nm is attenuated by oxygen B band absorption. The fluorescence line height (FLH) at 680 nm for [Chla] = 10 mg/m^3^, i.e., the difference between the observed measurement and the baseline, is shown in Figure 2a. The FLH algorithm proposed here can be applied to hyperspectral radiometric sensors onboard future satellites, such as the PACE mission [25].

**Figure 3.**
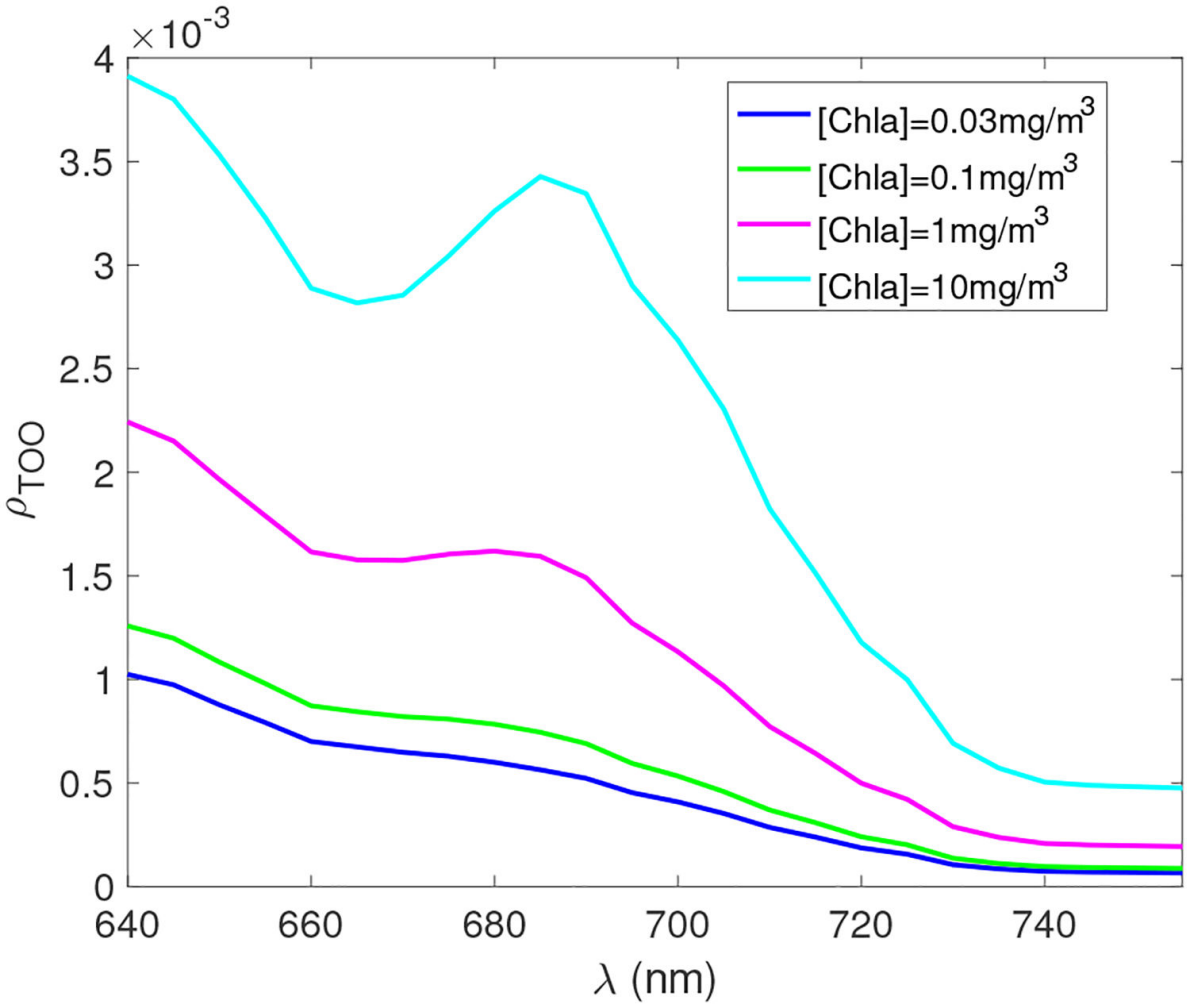
The nadir viewing reflectance at TOO as a function of wavelengths. The aerosol optical depth at 550 nm is 0.1 and the solar zenith angle is 30°.

The signal at TOA is dominated by the atmospheric scattering contribution such that the relative strength of chlorophyll a fluorescence is weak. This is not the case, however, for sensors in ocean waters. Figure 3 shows the nadir viewing reflectance at the top of the ocean (TOO) just below ocean surface as a function of wavelength. The reflectance at 680 nm varies from 0.0005 to 0.0035 for different [Chla] values. The spectral range of the fluorescence signals is approximately between 660 nm and 720 nm, which is primarily determined by *λ*_*C*,0_ = 685 nm and *σ*_*C*_ = 10.6 nm used in the simulation.

**Figure 4.**
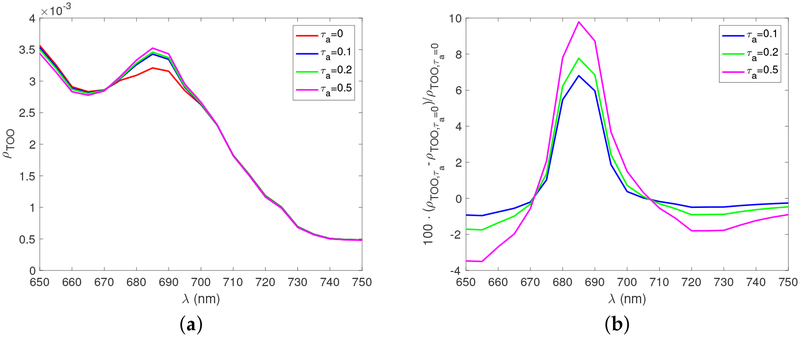
(**a**) The nadir viewing reflectance at TOO as a function of wavelengths for different aerosol optical depths. [Chla] = 10.0 mg/m^3^ and the solar zenith angle is 30°. (**b**) The percentage differences of fluorescence signals for different aerosol optical depths.

The quantum yield is strongly affected by the atmospheric conditions through their influence on IPAR. Figure 4a shows the nadir viewing reflectance at TOO for different aerosol optical depths at the reference wavelength of 550 nm. Figure 4b shows the relative signal variation with respect to *τ*_*a*_ = 0, which corresponds to a Rayleigh scattering only atmosphere. The fluorescence signal at TOO increases as the aerosol optical depth increases, which is somewhat counterintuitive. A detailed analysis revealed that this is a combined effect of a larger quantum yield value due to the decreased IPAR in Equation (1) and a smaller *E*_*o*_ term in Equation (5). The effect of larger quantum yield dominates the effect so that the fluorescence signal is larger for smaller IPAR values. This phenomenon has been observed by the Geostationary Ocean Color Imager [5]. The relative difference is from 6% to 10 % for optical depth ranging form *τ*_*a*_ = 0.1 to 0.5.

**Figure 5.**
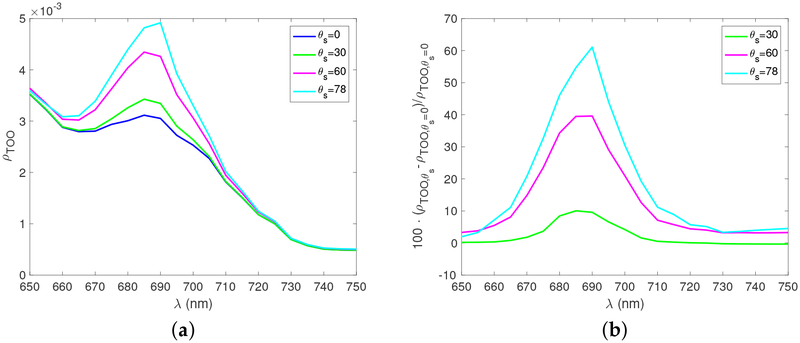
(**a**) The nadir viewing reflectance at TOO as a function of wavelengths for different solar zenith angles. [Chla] = 10.0 mg/m^3^ and the aerosol optical depth at 550 nm is 0.1. (**b**) The percentage differences of fluorescence signals at TOO for different solar zenith angles.

The solar zenith angle is a primary factor which impacts the irradiance field in the ocean. Figure 5a shows the nadir viewing reflectance at TOO for different *θ*_*s*_ values. The relative difference with respect to the case of *θ*_*s*_ = 0° is shown in Figure 5b. As the solar zenith angle increases, IPAR in ocean water decreases and hence the fluorescence signal increases for the same reason indicated by Figure 4b [5]. The fluorescence signal has variations from 10% to 60% when solar zenith increases from 0 to 78°.

## 4. Discussion

Our treatment of photochemical and nonphotochemical quenching processes in chlorophyll fluorescence relies on the fidelity of Equation (1), a model developed by Morrison et al. [1,8]. The quantum yield quenching model (Equation (1)) was used in satellite remote sensing of quantum yield which reveals striking correlation with soluble iron deposition in ocean [3]. The same concept was also applied to geostationary satellite observation to study daily to seasonal dynamics in phytoplankton photophysiology [5]. Both Behrenfeld et al. [3] and O’Malley et al. [5] used a semianalytical fluorescence model, which involved several approximations. The associate uncertainty of these simplifications has to be evaluated with rigorous radiative transfer simulation. Our newly developed model serves well on this purpose. It can also be used to generate synthetic dataset for testing future satellite algorithms which retrieves chlorophyll and/or phytoplankton physiology parameters

In vertically inhomogeneous water columns, the vertical location of phytoplankton has significant impacts on the water leaving signal and consequently on retrieved [Chla] values [48]. The effects of vertical distribution of phytoplankton to the water leaving fluorescence signal were not studied in this paper but will be the subject for a future study. The fluorescence signal variation at TOA is much suppressed by the dominating effect of atmosphere contribution to the radiation field. Nevertheless, the FLH has been detected and retrieved by MODIS, though nonphysical negative FLH data have been obtained in complex waters. This can be improved by using hyperspectral measurements made by the PACE ocean color instrument. One possible way to achieve this is to derive the fluorescence line height using a combination of 640 nm, 680 nm, 710 nm, and 745 nm.

The radiative transfer model can also be used to study the impacts of system configurations to the retrieval algorithms, for example, vertical distribution of phytoplankton blooms and atmospheric particles like aerosols and clouds. Recently a new trend in ocean color remote sensing is to inverse system parameters using nonlinear least squares fitting procedures, in which radiative transfer models are called dynamically to fit radiometric measurements [49–51]. Our model can be used for fitting fluorescence signals by varying phytoplankton physiology parameters in the same mathematically frame. Moreover, our radiative transfer model can be used to explore the information content regarding desired retrieval parameters [52].

The physiology parameters *q*_*I*_, *E*_*T*_, and *E*_*k*_ in Equation (1) depend on phytoplankton species and environmental factors including temperature, nutritions, etc. [8]. There is a lack of understanding in the literature on how these parameters varies with different environmental factors, which remains a future research direction in the marine biological community. In addition, we used the “average” bio-optical models of the inherent optical properties outlined in Section 3. It has been demonstrated that large natural variations exist in these relations [53]. To resolve the natural variations, we could use specific models in which regional and seasonal variability is included [54].

## 5. Conclusions

We report an exact radiative transfer solution that includes both photochemical and nonphotochemical quenching effects. Major gas absorptions in the atmosphere are considered in order to accurately simulate the scalar irradiance in ocean waters, which in turn regulates the quantum yield due to the quenching processes. Moreover, the scalar irradiance in ocean waters changes the inelastic source terms for fluorescence directly. The actual fluorescence signal depends on the compound effects of these two effects. The utility of this model is illustrated by using it to simulate the effect of photochemical and nonphotochemical quenching on radiance measured at TOA for an upcoming satellite and at TOO for an in situ sensor. Besides the obvious dependence on the chlorophyll a concentration, the fluorescence signal at TOO also varies for different aerosol optical depths and solar zenith angles. The relative variation of fluorescence ranges from 5% to 60% under different illumination conditions.
